# A Multi-Parametric Imaging Investigation of the Response of C6 Glioma Xenografts to MLN0518 (Tandutinib) Treatment

**DOI:** 10.1371/journal.pone.0063024

**Published:** 2013-04-26

**Authors:** Jessica K. R. Boult, Jennifer Terkelsen, Simon Walker-Samuel, Daniel P. Bradley, Simon P. Robinson

**Affiliations:** 1 Division of Radiotherapy and Imaging, The Institute of Cancer Research and Royal Marsden NHS Trust, Sutton, Surrey, United Kingdom; 2 Biomedical Imaging Group, Millennium: The Takeda Oncology Company, Cambridge, Massachusetts, United States of America; Wayne State University, United States of America

## Abstract

Angiogenesis, the development of new blood vessels, is essential for tumour growth; this process is stimulated by the secretion of numerous growth factors including platelet derived growth factor (PDGF). PDGF signalling, through its receptor platelet derived growth factor receptor (PDGFR), is involved in vessel maturation, stimulation of angiogenesis and upregulation of other angiogenic factors, including vascular endothelial growth factor (VEGF). PDGFR is a promising target for anti-cancer therapy because it is expressed on both tumour cells and stromal cells associated with the vasculature. MLN0518 (tandutinib) is a potent inhibitor of type III receptor tyrosine kinases that demonstrates activity against PDGFRα/β, FLT3 and c-KIT. In this study a multi-parametric MRI and histopathological approach was used to interrogate changes in vascular haemodynamics, structural response and hypoxia in C6 glioma xenografts in response to treatment with MLN0518. The doubling time of tumours in mice treated with MLN0518 was significantly longer than tumours in vehicle treated mice. The perfused vessel area, number of alpha smooth muscle actin positive vessels and hypoxic area in MLN0518 treated tumours were also significantly lower after 10 days treatment. These changes were not accompanied by alterations in vessel calibre or fractional blood volume as assessed using susceptibility contrast MRI. Histological assessment of vessel size and total perfused area did not demonstrate any change with treatment. Intrinsic susceptibility MRI did not reveal any difference in baseline R_2_* or carbogen-induced change in R_2_*. Dynamic contrast-enhanced MRI revealed anti-vascular effects of MLN0518 following 3 days treatment. Hypoxia confers chemo- and radio-resistance, and alongside PDGF, is implicated in evasive resistance to agents targeted against VEGF signalling. PDGFR antagonists may improve potency and efficacy of other therapeutics in combination. This study highlights the challenges of identifying appropriate quantitative imaging response biomarkers in heterogeneous models, particularly considering the multifaceted roles of angiogenic growth factors.

## Introduction

Tumour growth is dependent upon angiogenesis, the development of new blood vessels, to provide a nutritive blood supply [1]. This process is stimulated by the secretion of numerous growth factors by tumour cells, endothelial cells and tumour associated macrophages [2]. Tumour vasculature is structurally irregular, with vessels being more tortuous, fragile, dilated and hyperpermeable in comparison to normal blood vessels [3]. Considering their critical role in tumour development and progression, angiogenic growth factors are the target for many novel cancer therapeutics.

The platelet derived growth factor (PDGF) family of growth factors exert their cellular effects by binding to, and activating, the structurally related tyrosine kinase receptors PDGFRα and PDGFRβ [4]. PDGF/PDGFR signalling is involved in vessel maturation and the development of an ordered vascular hierarchy. It has also been shown to stimulate angiogenesis [5] and upregulate the expression of other angiogenic factors, such as vascular endothelial growth factor (VEGF) [6]. Paracrine signalling between PDGF-BB, expressed by vascular endothelial cells, and PDGFRβ, expressed by mural cells, plays a central role in the recruitment of pericytes and the stabilisation of blood vessels [7], [8]. PDGF/PDGFR signalling also acts on many other components of a tumour and its microenvironment, including the autocrine and paracrine stimulation of tumour cells resulting in enhanced cell growth and motility [4], and the regulation of interstitial fluid pressure (IFP) [9]. Targeting PDGF/PDGFR signalling thus represents an attractive anti-tumour strategy.

MLN0518 (tandutinib, Millennium Pharmaceuticals Inc, Cambridge, MA, USA) is a potent, ATP-competitive and reversible inhibitor of type III receptor tyrosine kinases that crosses the blood brain barrier and demonstrates activity against PDGFRα/β, FLT3 and c-KIT in the submicromolar range *in vitro* [10].

The identification and investigation of biomarkers of treatment response is imperative in the development of molecularly targeted anti-cancer drugs [11]. Magnetic resonance imaging (MRI) and quantitative immunohistochemical techniques provide an array of biomarkers to assess the response of tumours to such agents. Intrinsic and extrinsic contrast agents can be deployed in MRI to assess key hallmarks of tumour vascular pathophysiology. For example, dynamic contrast-enhanced (DCE) MRI, which uses a contrast agent capable of leaking out of hyperpermeable tumour blood vessels, is now routinely requested and deployed in both clinical and experimental pharmacological tumour investigations, and is particularly powerful for the assessment of agents targeted to tumour vasculature [12]. Susceptibility contrast MRI uses an intravascular contrast agent that can inform on blood vessel calibre and fractional tumour blood volume [13]. Intrinsic susceptibility MRI (also referred to as blood oxygen level dependent (BOLD) MRI) exploits the paramagnetic nature of deoxyhaemoglobin, and following administration of a high oxygen gas, can inform about the functional (haemodynamic) nature of the tumour vasculature [14]. The addition of histological readouts of perfusion, blood vessel density, vessel maturation and hypoxia provides appropriate qualification of the imaging biomarkers investigated.

In this study, a multi-parametric MRI and histopathological approach was used to interrogate the vascular structural and haemodynamic response, and the hypoxic changes in C6 glioma xenografts in response to MLN0518.

## Materials and Methods

### Cell Culture

C6 rat glioma cells (European Collection of Cell Cultures, Salisbury, UK) were maintained in Ham's F-10 medium supplemented with 10% (v/v) foetal bovine serum and 5 mM L-glutamine (all Invitrogen, Life Technologies, Paisley, UK).

### Ethics Statement

All procedures undertaken at the Institute of Cancer Research were approved by the Institute of Cancer Research Ethical Review Committee and with the authority of Personal and Project Licences issued by the UK Home Office under the Animals (Scientific Procedures) Act 1986, and in accordance with the United Kingdom National Cancer Research Institute guidelines for the welfare of animals in cancer research [15]. All experiments performed at Millennium for this study were conducted under an approved Millennium Institutional Animal Care and Use Committee (IACUC) protocol, and animals were housed and handled in accordance with the Guide for the Care and Use of Laboratory Animals. Millennium is an Association for Assessment and Accreditation for Laboratory Care International designated institution and adheres to the highest standards of ethical treatment in animals. Tumour propagation and MRI were performed under general anaesthesia at both institutions and every effort was made to minimise suffering at all times.

### Tumour Propagation

C6 cells (2×10^6^) were injected subcutaneously into the flanks of female NCr nude mice under isoflurane anaesthesia. Tumour size was monitored using callipers and growth curves were obtained for individual tumours using the formula for ellipsoid volume (L×W^2^)/2, where L and W were the two largest dimensions of the ellipsoid. Once tumours reached 200±100 mm^3^ they were randomised to receive either 20 mg/kg MLN0518 or vehicle (5% dextrose) subcutaneously twice daily for either three or ten days.

### Magnetic Resonance Imaging

Following either three or ten days treatment with 20 mg/kg MLN0518 or vehicle, mice underwent ^1^H MRI performed on either a 7T Bruker horizontal bore microimaging system (Ettlingen, Germany) using a 3 cm birdcage coil or a Varian Inova 7T horizontal bore system (Palo Alto, CA, USA) using a 63 mm quadrature coil. For susceptibility contrast and intrinsic susceptibility MRI, anaesthesia was induced with a 10 ml/kg intraperitoneal injection of fentanyl citrate (0.315 mg/ml) plus fluanisone (10 mg/ml (Hypnorm; Janssen Pharmaceutical Ltd. High Wycombe, UK)), midazolam (5 mg/ml (Hypnovel; Roche, Burgess Hill, UK)) and sterile water (1∶1∶2). For dynamic contrast-enhanced MRI, anaesthesia was induced with 3–4% isoflurane in air (2L/min) and maintained at 1–2%. For the contrast-enhanced protocols a lateral tail vein was cannulated with a 27G butterfly catheter (Venisystems, Hospira, Royal Leamington Spa, UK) to enable the remote administration of either USPIO particles (ferumoxtran-10, Sinerem®; Guerbet, Villepinte, France) or Gd-DTPA (Magnevist™; Schering, Berlin, Germany). Core body temperature was maintained by warm air blown through the magnet bore.

#### Susceptibility Contrast and Diffusion-Weighted MRI

A rapid acquisition with relaxation enhancement (RARE) T_2_-weighted sequence (field of view = 3×3 cm, 128×128 matrix, 20×1 mm thick contiguous axial slices, 4 averages, effective echo time (T_Eeff_) = 36ms, repetition time (T_R_) = 5000 ms) was first used for localisation of the tumour and measurement of tumour volume. Multi-gradient echo (MGE, T_R_ = 300 ms, 8 echo times (T_E_) ranging from T_E_ = 6.2 to 28.2 ms, flip angle = 45°, 16 averages), spin echo (SE, T_R_ = 3000 ms, 2 echo times of 8 and 80 ms, flip angle = 90°, 2 averages) and diffusion-weighted spin echo (DWSE, 6 b-values ranging from 6 to 504 s/mm^2^ in the read direction, gradient strength = 0 to 0.1T/m, gradient duration = 5 ms, gradient spacing = 25 ms, T_R_ = 1000 ms, T_E_ = 36 ms) images were acquired for quantification of R_2_*, R_2_ and ADC, respectively, from 3 contiguous 1mm thick axial slices through the largest extent of the tumour, with a 64×64 matrix over a 3×3 cm field of view [16]. Following acquisition of these data, USPIO particles were administered as a bolus (200 μmol Fe/kg i.v.) and allowed to circulate for two minutes in order to equilibrate before a second set of MGE and SE images were acquired. The time taken to perform the entire MRI protocol was typically 100 minutes per mouse. This protocol was performed on tumour bearing mice treated with vehicle (n = 6 at 3 days, n = 5 at 10 days) or MLN0518 (n = 6 at 3 and 10 days). Separate cohorts of mice were used for the two time points to enable the provision of pathological correlates.

#### Intrinsic Susceptibility MRI

A morphological RARE sequence was used for tumour localisation, following which MGE images (field of view = 3×3 cm, matrix size 128×128, T_R_ = 200 ms, 8 echo times ranging from T_E_ = 6.2 to 28.2 ms, flip angle = 45°, 16 averages) were acquired for quantification of R_2_* from three contiguous axial slices through the largest extent of the tumour. Carbogen (5% CO_2_/95% O_2_) was then continuously administered via a nose piece at 1L/min; following a transition period of at least 10 minutes a second set of MGE images were acquired. This protocol was performed on vehicle treated (n = 5) and MLN0518 treated (n = 6) animals.

#### Dynamic Contrast-Enhanced MRI

Fast spin echo T_2_-weighted coronal pilot scans were acquired to locate the tumour. DCE MRI data were acquired from a single 2 mm thick sagittal slice through the centre of the tumour using an inversion recovery fast low angle shot (FLASH) imaging sequence (T_E_ = 1.3 ms, T_R_ = 4 ms, inversion times of 0.005, 0.01, 0.05, 0.1, 0.5, 1, 2, 5 and 15 s, flip angle = 20°, 2 averages), using a 64×128 matrix (subsequently zero-filled to 128×128) over a 6×6 cm field of view). Gd-DTPA (0.1mmol/kg) was manually injected i.v. over 5 seconds after 10 baseline scans were acquired, and image acquisition continued for a further 70 scans [17]. This protocol was performed on vehicle treated (n = 8) and MLN0518 treated (n = 6) animals.

### MRI Analysis

Parameter estimation was undertaken using a Bayesian maximum *a posteriori* algorithm, which took into account the Rician distribution of noise in magnitude MR data in order to provide unbiased parameter estimates [18]. ADC was determined from DWSE MRI data. Estimates of the MRI transverse relaxation rates R_2_* and R_2_ were determined from the susceptibility contrast MRI data. Changes in each measured relaxation rate following delivery of USPIO (ΔR_2_* and ΔR_2_) were also evaluated, from which fractional blood volume (fBV, %) and vessel size index (R_v_, µm) were estimated [19], [20]. Estimates of R_2_* and ΔR_2_* from the intrinsic susceptibility MRI data were calculated in the same manner, to reflect changes induced by carbogen breathing. All data were fitted on a pixel-by-pixel basis using in-house software (ImageView developed in IDL, ITT Visual Information Systems, Boulder, CO, USA). The median value of each parameter in each tumour was determined. For the DCE MRI data whole tumour ROIs were drawn and no regional segmentation performed. The concentration of Gd-DTPA was determined from the basal T_1_ and the previously measured relaxivity at 7T of 3.2/mM/s. Parametric IAUGC (initial area under the gadolinium concentration curve) maps were subsequently generated for the whole image [17].

### Histological Analysis

Tumour bearing mice were administered 60 mg/kg hypoxia marker pimonidazole hydrochloride (Hypoxyprobe, Burlington, MA, USA) in phosphate-buffered saline (PBS) i.p. After at least 45 minutes 15 mg/kg of the perfusion marker Hoechst 33342 (Sigma-Aldrich, Poole, UK) in PBS was injected intravenously through a lateral tail vein [21]. After 1 minute tumours were rapidly excised and cut in half; one half was snap frozen and stored in liquid nitrogen, the other was fixed in 10% formalin in saline (v/v) and embedded in paraffin wax.

Hoechst 33342 fluorescence signals from whole frozen tumour sections (10 μm, two per tumour, 15 animals per treatment group) were recorded at 365 nm using a motorised scanning stage (Prior Scientific Instruments, Cambridge, UK) attached to a BX51 microscope (Olympus Optical, London, UK) driven by CellP (Soft Imaging System, Münster, Germany). In addition, individual images from localised regions of each section were acquired. From these images the vascular morphology was visualised and the largest vessel diameter measured using CellP [22].

The same sections were then processed for the simultaneous detection of pimonidazole adducts and vascular endothelial marker CD31 [21]. Sections were first incubated with 2% (w/v) BSA/5% (v/v) goat serum in PBS for 1 hour to block any non-specific antibody binding, and then with goat anti-mouse CD31 antibodies (MEC 13.3, BD Biosciences, Oxford, UK, 1∶100) overnight at 4°C. Sections were washed with 0.1% (v/v) Tween-20 in PBS, then incubated with Alexa 546-conjugated goat anti-rat secondary antibodies (Invitrogen, 1∶500) and Hypoxyprobe-1 plus FITC-conjugated mouse monoclonal antibodies (Hypoxyprobe Inc., 1∶200) at 37°C for 4 hours, protected from light. Following washing the sections were mounted in Vectashield mounting medium (Vector Laboratories, Peterborough, UK) with a coverslip. Regions of pimonidazole adduct formation were detected at 450–490 nm and CD31 expression detected at 510–560 nm using the same fluorescence microscope system and stage co-ordinates. Composite images of whole tumour sections were recorded, allowing the pimonidazole and CD31 images to be subsequently overlaid on the Hoechst 33342 images. Fluorescent particles were detected above a constant threshold and the area of the tumour section with Hoechst 33342, pimonidazole adduct or CD31 fluorescence was determined and expressed as a percentage of the whole tumour section. Co-localisation of Hoechst 33342 (perfused vessels) and CD31 (total vessels) was also assessed and expressed as a percentage total vessels perfused and a total perfused vessel area.

Alpha smooth muscle actin (α-SMA) immunohistochemistry was performed on 5 µm formalin-fixed paraffin-embedded (FFPE) sections (n = 12 per treatment group) using mouse monoclonal anti-α-SMA antibodies (1A4, Sigma-Aldrich, Poole, UK, 1∶500) and EnVision Dual link HRP-labelled polymer (Dako, Ely, UK). Composite images of whole tumour sections were acquired using the same microscope system under light. Whole tumour images were acquired to determine areas of α-SMA staining; positively stained vessels were counted from 6 fields at 200× magnification per tumour section [23].

FFPE sections were also stained with haematoxylin and eosin (H&E) for the assessment and quantification of necrosis (n = 14 per treatment group). Composite images of whole tumour sections were recorded, and the degree of tumour necrosis was quantified and expressed as a percentage of the whole tumour section area [24].

### Statistical Analysis

Results are presented as the mean ±1 standard error of the mean (s.e.m.). Significance testing used a Student's unpaired t-test or Mann-Whitney test with a 5% confidence level to compare vehicle and MLN0518 treated tumours at the study endpoint.

## Results

**Figure 1:**
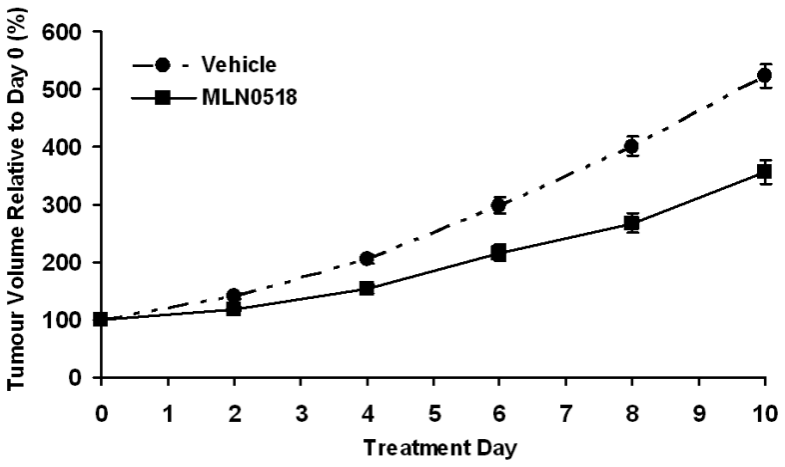
C6 tumour growth is slowed by MLN0518 treatment. The growth rate of tumours in mice treated with 20 mg/kg MLN0518 was significantly slower than tumours in vehicle treated mice over 10 days. Tumour doubling times were calculated on an individual tumour basis (n = 15 per treatment group). Mean ±1s.e.m.

Mean growth curves for tumours grown in mice receiving 20 mg/kg MLN0518 or vehicle, expressed as a percentage of the tumour volume immediately prior to the first dose, are shown in Figure 1. Individual growth curves were obtained for each tumour and the volume doubling time calculated for the duration of treatment [21]. The mean growth rate of tumours in mice treated with MLN0518 for ten days (mean doubling time 5.7±0.2 days) was significantly slower than tumours in vehicle treated mice (4.5±0.3 days, p<0.01).

**Figure 2:**
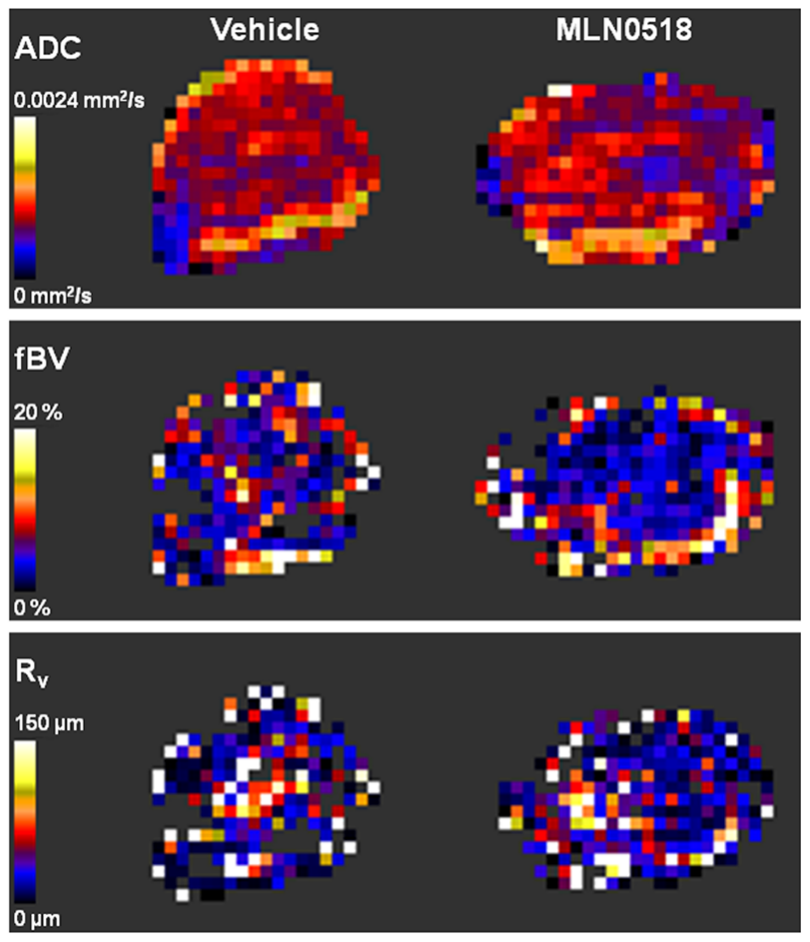
Diffusion-weighted and susceptibility contrast MRI of C6 tumours treated with MLN0518. Parametric maps of apparent diffusion coefficient (ADC, top panel), fractional blood volume (fBV, middle panel) and vessel size index (R_v_, bottom panel) from C6 xenografts in mice treated with vehicle or 20 mg/kg MLN0518 for 10 days show no clear differences between vehicle and treated tumours. Representative maps are shown.

At the end of the ten day treatment period tumours were investigated by MRI. Representative parametric maps of apparent diffusion coefficient (ADC), a measure of the magnitude of Brownian water diffusion within tissue and an imaging biomarker of cellularity, fractional blood volume (fBV) and vessel size index (R_v_) are shown in Figure 2. A summary of the data determined for each quantitative MR imaging biomarker is presented in Table 1. Susceptibility contrast and diffusion-weighted MRI demonstrated no significant differences in the imaging biomarkers fBV, R_v_ and ADC and between vehicle and MLN0518 treated tumours. Quantification of total Hoechst 33342 uptake and vessel size measurements taken from Hoechst 33342 stained tumour sections also showed no significant differences in total perfusion and vessel size, respectively, with MLN0518 treatment (Table 2).

**Table 1:** Summary of the quantitative MRI biomarkers acquired from diffusion-weighted and susceptibility contrast MRI of C6 xenografts in mice treated with vehicle or 20 mg/kg MLN0518 for 10 days.

MRI Biomarker	Vehicle	20mg/kg MLN0518
ADC (x10^−6^mm^2^/sec)	917±25	921±43
Fractional blood volume (%)	3.7±0.5	4.5±0.6
Vessel size index (µm)	23.8±2.7	21.9±4.7

ADC  =  apparent diffusion coefficient. Mean of median parameter values from each tumour ± 1s.e.m. (n≥5 per treatment group).

**Table 2:** Summary of the histological biomarkers assessed in C6 xenografts in mice treated with vehicle or 20 mg/kg MLN0518 for 10 days.

Histological Biomarker	Vehicle	20mg/kg MLN0518
Hoechst vessel size (µm)	29.1±0.4	28.1±0.3
Perfused vessels (%)	42.5±2.3	31.7±2.0 **
Perfused vessel area (%)	1.9±0.2	1.2±0.1 **
Pimonidazole (%)	10.9±1.0	7.8±0.6 *
α-smooth muscle actin (vessels/field)	2.8±0.3	2.0±0.3 *
Necrosis (%)	41.2±2.2	41.1±2.4

Values are mean ± 1s.e.m. Two frozen sections per tumour were assessed for fluorescence microscopy (n = 15 per treatment group) and a single FFPE section per tumour for α-smooth muscle actin and necrosis assessment (n≥12 per treatment group). ^*^ p<0.05, ^**^ p<0.01.

**Figure 3:**
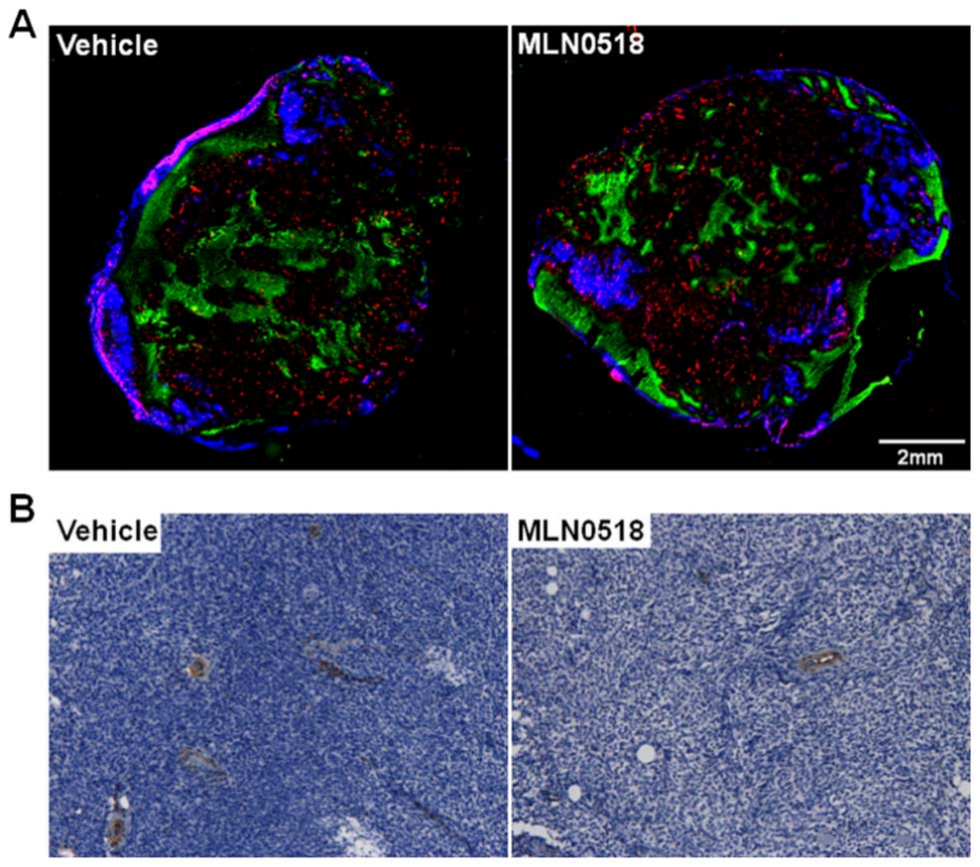
Histological assessment of tumour response to MLN0518. **A.** Tumour sections stained for the perfusion marker Hoechst 33342 (blue), endothelial marker CD31 (red) and pimonidazole adduct formation, a marker of hypoxia (green) demonstrate that the hypoxic area was lower in tumours treated with 20 mg/kg MLN0518 for 10 days than vehicle treated controls. The percentage of the total vessels perfused and the overall perfused vessel area was also lower in treated versus control tumours. Representative composite images are shown. **B.** Alpha smooth muscle actin (α-SMA) immunohistochemistry demonstrates a significant reduction in α-SMA positive blood vessels in MLN0518 treated tumours compared to controls. Magnification ×200.

Assessment of the co-localisation of Hoechst 33342 and CD31 (Figure 3A, Table 2) revealed that the percentage of the total vessels perfused at the time of Hoechst 33342 injection was 17.1% lower in MLN0518 than vehicle treated tumours (p<0.01), which equated to a 37.5% reduction in an overall perfused vessel area in treated versus control tumours (p<0.01). However, neither the Hoechst 33342 perfused area nor the total CD31 positive endothelial cell area significantly differed between vehicle and MLN0518 treated tumours (quantification not shown). Despite having a lower perfused vessel area, MLN0518 treated tumours were 25.3% less hypoxic than control tumours as assessed by pimonidazole adduct formation (p<0.05). Immunohistochemistry demonstrated a significant reduction in alpha smooth muscle actin positive blood vessels in MLN0518 treated tumours compared to vehicle treated tumours (p<0.05, Table 2, Figure 3B). H&E staining revealed no difference in necrosis between the groups (Table 2).

In light of the MLN0518-induced alteration in perfused vessel area, vascular maturation and hypoxia, intrinsic susceptibility MRI was performed on a separate cohort of C6 tumour bearing mice treated with either vehicle or 20 mg/kg MLN0518. There was no difference in the baseline relaxation rate R_2_* or in the change in R_2_* induced by carbogen breathing between vehicle and MLN0518 treated tumours. Similarly, the percentage of voxels with a negative ΔR_2_*, representing oxygenation of the haemoglobin, was not significantly different between the treatment groups (Table 3).

**Table 3:** Summary of the quantitative MRI biomarkers acquired from intrinsic susceptibility MRI of C6 xenografts in mice treated with vehicle or 20 mg/kg MLN0518 for 10 days.

MRI Biomarker	Vehicle	20mg/kg MLN0518
Baseline R_2_* (s^−1^)	67.2±4.2	84.7±14.5
ΔR_2_* (s^−1^)	3.2±2.8	4.7±2.2
ΔR_2_* <0 (%)	36.2±2.8	29.9±3.1
ΔR_2_* >0 (%)	40.8±4.4	41.8±3.8

Mean of median R_2_* and ΔR_2_* values from each tumour ± 1s.e.m. (n≥5 per treatment group). The proportion of voxels in which R_2_* changed significantly, either negatively (ΔR_2_* <0) or positively (ΔR_2_* >0), with carbogen breathing are also shown.

**Figure 4:**
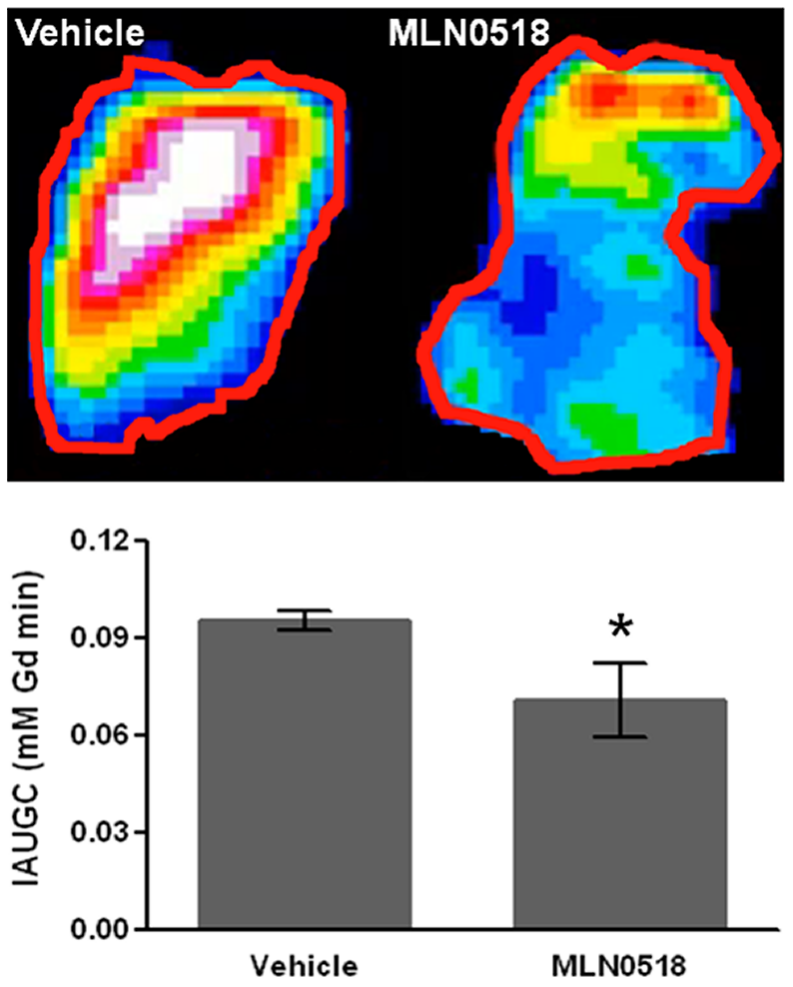
Dynamic contrast-enhanced MRI is sensitive to the response of C6 tumours to MLN0518. Representative parametric maps and quantification of initial area under the gadolinium concentration curve (IAUGC) demonstrate a reduction in tumour blood vessel permeability/flow in mice treated with 20 mg/kg MLN0518 for 3 days compared to controls. Mean parameter values from each tumour ± 1s.e.m. (n≥6 per treatment group), * p<0.05.

Tumour growth was also delayed by treatment with MLN0518 over three days. As was observed after ten days treatment, MLN0518 treated tumours demonstrated a 30.8% smaller overall perfused vessel area and were 37.0% less hypoxic than vehicle treated tumours. Susceptibility contrast MRI did not reveal any change in R_v_, fBV or ADC at this time point (data not shown). Dynamic contrast-enhanced MRI performed following three days treatment with MLN0518 or vehicle revealed that the initial area under the gadolinium concentration curve (IAUGC) was significantly lower in tumours in MLN0518 treated mice than in control tumours (p<0.05, Figure 4).

## Discussion

PDGF/PDGFR signalling is an attractive target for anti-cancer therapy; it has a pleiotropic role on the tumour microenvironment, principally in vascular maturation, but it also has direct effects on tumour cells and hypoxia [4], [7], [8], [25]. Glioblastoma multiforme (GBM), one of the most vascularised human tumours [26], expresses both PDGF receptors and their ligands and demonstrates altered PDGF pathway signalling [27]. Typically, PDGFRα is highly expressed by tumour cells and PDGFRβ is preferentially expressed by vascular endothelial and mural cells within tumours. However, PDGFRβ has been shown to be expressed in 50% of tumour cells in addition to 65% of peritumoural endothelial cells in GBM [28]. Co-expression of PDGF and both receptors is an indication that autocrine/paracrine signalling plays an important role in glial tumour establishment and development [29], [30]. Consequently, the C6 glioma xenograft model was utilised in this study combining multi-parametric MRI and quantitative histopathology to assess the response to MLN0518.

Tumour growth in mice bearing C6 xenografts was suppressed by twice daily treatment with 20 mg/kg MLN0518. This growth inhibition was accompanied by a significant reduction in the number of α-SMA positive tumour blood vessels and a reduction in the percentage of tumour vessels perfused at the time of tumour excision, demonstrating that target inhibition had been achieved and that inhibition of PDGFR signalling with MLN0518 has an anti-vascular effect. However, the changes in growth rate and vascular histological parameters following treatment with MLN0518 were not reflected in the biomarkers afforded by susceptibility contrast or intrinsic susceptibility MRI.

No difference in vessel calibre was observed between control and MLN0518 treated tumours using susceptibility contrast MRI (R_v_) or by histological vessel size measurement. Considering the role of PDGF in vascular maturation and developing a vascular hierarchy, coupled with evidence that the number of tumour blood vessels possessing a smooth muscle element to their wall was lower in treated tumours than controls, an increase in vessel calibre following MLN0518 treatment was anticipated [31]. However, it is possible that the pericytes present in these tumours are structurally or functionally abnormal [32] and therefore any reduction in pericyte coverage does not elicit a significant effect on vessel calibre. Regions of high fBV detected by MRI were spatially correlated with areas of high Hoechst 33342 uptake, typically at the tumour rim; however neither fBV nor total Hoechst 33342 perfused area demonstrated any significant differences with treatment. A reduction in fBV may have been anticipated when considering the role of MLN0518 as a vascular targeting agent, causing an overall loss of vascular integrity and anti-angiogenic effects [5], [6], [33], but given the anti-maturation activity specifically associated with PDGFR inhibition [7], [8] the relative increase in more immature vessels post-treatment could equate to increased fBV. These opposing predicted effects of PDGFR inhibition on tumour blood volume may explain the lack of an overall change in fBV and Hoechst 33342 perfused area. Perfused vessel area was lower in treated tumours, it could therefore be concluded that there is increased vascular redundancy in MLN0518 treated tumours. This elevation in unperfused vessels may be as a result of vascular collapse following a loss in vascular integrity as a result of PDGFR inhibition [33].

Quantification of ADC, a measure of the magnitude of Brownian water diffusion within tissue and an imaging biomarker of cellularity, demonstrated no difference between control and MLN0518 treated tumours, indicating that cellular integrity was maintained during the anti-tumour effect of MLN0518 [34]. This was corroborated by quantification of necrosis from H&E stained tissue sections, demonstrating no difference in the percentage necrosis between the groups.

Intrinsic susceptibility MRI exploits the presence of paramagnetic deoxyhaemoglobin in erythrocytes and can be used to assess the change in the oxygenation of haemoglobin induced by carbogen breathing. However, global tumour ΔR_2_* induced by carbogen breathing and the proportion of voxels in which ΔR_2_* was negative, indicating a replacement of paramagnetic deoxyhaemoglobin with diamagnetic oxyhaemoglobin and therefore indicative of perfused vasculature, did not change with MLN0518 treatment.

The presence of tumour hypoxia in solid tumours is associated with resistance to radiation therapy and chemotherapy, the selection of more invasive and metastatic clones, and poor patient prognosis [35], [36]. Assuming that the oxygenation of haemoglobin is proportional to the arterial blood oxygen pressure, and therefore in equilibrium with tissue oxygen tensions, static intrinsic susceptibility MRI measurements of R_2_* is a putative method with which to assess tumour hypoxia [14]. Hypoxia assessed by histological detection of pimonidazole adduct formation was significantly reduced by MLN0518 treatment. However, baseline R_2_* did not differ between the treatment groups, suggesting that intrinsic susceptibility MRI was not sensitive enough to assess this difference. The reduction in hypoxia in the treated tumours, despite a decrease in the perfused vessel area, suggests that the degree of hypoxia is related to the anti-tumour efficacy of MLN0518, with the slower tumour growth rate equating to a smaller percentage of hypoxic tumour tissue.

Unlike MLN0518 many new clinically approved targeted receptor tyrosine kinase inhibitors, whilst improving overall survival as monotherapies and in combination [37], [38], have been shown in preclinical models to increase the hypoxic fraction in distant metastases [39]. PDGF/PDGFR signalling has been shown to be involved in evasive resistance to inhibitors of VEGF signalling, partly as a result of the increased periendothelial support provided by pericytes, which protects the endothelial cells from being targeted by VEGF/VEGFR inhibition [40]. This, coupled with the role of hypoxia in evasive resistance [41], suggests that treatment with MLN0518 may provide for an environment that supports a less resistant and malignant tumour phenotype and improve delivery, potency and efficacy of other therapeutics in combination or as a monotherapy.

Having established both histological and growth inhibitory evidence of response after ten days treatment with MLN0518, a more acute time point of three days was selected for further investigation into the time window of any measurable MRI biomarker response. A similar pattern of results were observed; tumour growth inhibition, reduced perfused vascular fraction and reduced hypoxia, but no observable change in fBV or R_v_. These results suggest that the effects of MLN0518 occur early and are maintained over a chronic treatment time course. DCE MRI is widely used to assess tumour vascular response to anti-vascular agents in oncology [42], we therefore investigated DCE MRI, using a clinically approved low molecular weight contrast agent, for the provision of more sensitive biomarkers of response to PDGFR inhibition in the C6 model. A significant reduction in tumour blood vessel permeability/flow was detected following treatment with MLN0518, illustrating that the drug has anti-vascular effects at this time point.

Treatment with MLN0518 demonstrated significant anti-tumour activity against C6 glioma xenografts, coupled with significant reductions in tumour hypoxia and perfused vessel area that, importantly, were associated with significant target inhibition. However, the corresponding imaging biomarkers afforded by susceptibility contrast and intrinsic susceptibility MRI failed to show the anticipated changes. This can be described as a false-negative imaging biomarker response [43]. We are therefore not recommending the deployment of these imaging techniques for the detection of response to PDGFR inhibition in this context; however DCE MRI appears to be more informative regarding the anti-vascular effects of PDGFR inhibition.

A limitation of this study is the use of an ectopic glioma xenograft model, which does not faithfully emulate the microenvironment, tumour-host stromal interactions and host vasculature factors that affect tumour growth and therapeutic response. Further studies should be performed in orthotopic or transgenic tumour models, in addition to subcutaneous models, to assess the suitability of MLN0518 and other PDGF antagonists for use in the treatment of glioma.

This study highlights the challenges of identifying quantitative imaging response biomarkers in heterogeneous models, particularly considering the multifaceted roles of angiogenic growth factors. For the assessment of tumour response to a novel therapeutic a rational selection of the imaging biomarker to use can be made taking into account the putative mechanism of action of the drug. However, experience shows that the most appropriate biomarker that will reveal objective tumour response cannot always be predicted. Alternative non-invasive imaging methods that may be more informative in the assessment of PDGFR inhibition in tumours may include measurement of interstitial fluid pressure (IFP) and magnetic resonance elastography (MRE).
