# Correction: A Multi-Parametric Imaging Investigation of the Response of C6 Glioma Xenografts to MLN0518 (Tandutinib) Treatment

**DOI:** 10.1371/annotation/37d4affb-9a93-44f3-8546-ad9cc07c7805

**Published:** 2013-09-12

**Authors:** Jessica K. R. Boult, Jennifer Terkelsen, Simon Walker-Samuel, Daniel P. Bradley, Simon P. Robinson

The name of an employer of the second and fourth authors was incorrectly given in the Competing Interests Statement. The Competing Interests Statement should read: "The authors Daniel P. Bradley and Jennifer Terkelsen are employees of Millennium: The Takeda Oncology Company. This does not alter the authors' adherence to all the PLOS ONE policies on sharing data and materials." 

